# Cardiovascular load and physical capacity in older workers engaged in physically demanding occupations

**DOI:** 10.1007/s00420-025-02161-8

**Published:** 2025-08-14

**Authors:** Viktoria Wahlström, Farhad Abtahi, Mikael Forsman, Liyun Yang, Pontus Öhrner, Andreas Tornevi, Albin Stjernbrandt, Charlotte Lewis, Lisbeth Slunga Järvholm

**Affiliations:** 1 Department of Epidemiology and Global Health, Umeå University, 901 87 Umeå, Sweden; 2 Division of Ergonomics, KTH Royal Institute of Technology, Stockholm, Sweden; 3 Institute of Environmental Medicine, Karolinska Institutet, Stockholm, Sweden; 4 Sports Medicine, Department of Community Medicine and Rehabilitation, Umeå University, Umeå, Sweden

**Keywords:** Construction workers, Kitchen workers, Cleaners, Assistant nurses, Occupational physical activity, Prolonged working life

## Abstract

**Objective:**

To measure and determine the occupational cardiovascular workload and cardiovascular fitness among older employees (50 +) in four physically demanding occupational groups.

**Methods:**

Health investigations, including aerobic capacity, were performed on 120 construction and kitchen workers, cleaners, and assistant nurses. Cardiovascular load was assessed over three working days using heart rate (HR) measurements. Data was processed for average loads (HR and Relative HR reserve (%HRR)) and measures describing variations in loads, such as time spent in different heart rate intensities.

**Results:**

Participants’ mean age was 57 (SD 4.1) years, and 63% of the participants were female. The mean %HRR over occupational groups was 24.9% (SD 6.8). Of the participants, 43% had an average cardiovascular load above 24.5%HRR and 11% over 33%HRR. Depending on the work sector, 22–37% of the worktime was spent in intensities over 30%HRR, and 2–4% was spent in cardiovascular intensities over 50%HRR. The average VO_2_max was 33.2 (SD 8.2) ml/kg/min for all, for men 39.0 (SD 7.0), and for women 29.8 (SD 6.9).

**Conclusion:**

We found a high mean cardiovascular load at work among the senior workers in all four work sectors, but low proportions of the worktime were spent in high intensity levels. Despite a high mean cardiovascular load at work, a high proportion of the workers had low cardiovascular fitness. Given the possible negative effects of occupational physical activity and to meet future demographic challenges, future interventions should aim to reduce physical loads and increase physical fitness in the workforce.

**Supplementary Information:**

The online version contains supplementary material available at 10.1007/s00420-025-02161-8.

## Background

Heavy physical work often involves working while standing or walking, manual handling, and working in uncomfortable body postures such as kneeling or forward bending. Contrasting the beneficial cardiovascular health effects provided by leisure time physical activity (LTPA) (Arem et al. 2015; Ekelund et al. 2019), studies suggest high levels of occupational physical activity (OPA) to be harmful and associated with increased risk of cardiovascular mortality (Coenen et al. 2018; Holtermann et al. 2021; Fontana et al. 2024). A hypothesized mechanism for the opposing effects of physical activity during leisure time and at work is that OPA typically lasts for many consecutive hours without enough recovery time and rarely reaches the intensity needed to improve cardiovascular fitness. Consequently, this could lead to an elevated long-term heart rate (HR) and higher blood pressure, and thereby, an increased risk of cardiovascular disease and mortality (Holtermann et al. 2018). This theory is supported by studies showing that high physical intensity at work is associated with a higher HR during the following night (Korshøj et al. 2021; Matias et al. 2022).

Most previous studies investigating occupational physical demands have used self-reported data to assess occupational physical workload (Coenen et al. 2018), but HR measurements can be used to assess physical workload with higher validity (Dias et al. 2023). HR increases linearly with increased oxygen uptake during continuous aerobic activities and is a feasible method to assess the cardiovascular load (Borg 1982; Dias et al. 2023). A common OPA assessment method is relative HR reserve (%HRR), which represents the cardiovascular load and accounts for variations in both resting HR and the estimated maximum HR (Korshøj et al. 2015; Merkus et al. 2019; Dias et al. 2023). For an 8-h working day, the International Labor Organization (ILO) suggests that the mean physical workload should not exceed 33 percent of the maximal oxygen uptake (VO_2_max) (Wultsch et al. 2012; Wu and Wang 2002). In previous studies using %HRR as the outcome variable, results are most often presented and discussed in relation to 33%HRR (Lunde et al. 2016; Lidegaard et al. 2018; Merkus et al. 2019). However, %HRR is not equivalent to %VO_2_max, and a workload of 34%VO_2_max is suggested to correspond to 24.5%HRR when expressed as for 8 h of work in young adults (Swain and Leutholtz 1997; Wu and Wang 2002). This corresponding limit is based on a laboratory study showing how different workloads affected the cardiovascular load in work shifts of different lengths (Wu and Wang 2002).

Demographic changes in Western societies result in aging populations and a reduction of the relative proportion of citizens of working age (Crawford et al. 2016; European Commission 2024). To meet the pressures of economies and welfare systems, increasing labor market participation among older workers has received political attention in recent years. Many European countries, Sweden included, have regulated their national pension schemes towards increased retirement age (OECD 2023). Although the proportion of physically demanding jobs has decreased, 43% of the workers in Sweden still report having such jobs (Arbetsmiljöverket 2022). In Europe, over 50% of low- or mid-skilled workers doubt they will be able to do the same job at 60 years of age, and a Danish study found that more than 25% of older workers with physically demanding occupations are expected to leave the labor market due to poor physical health and work difficulties (Crawford et al. 2016; Andersen et al. 2021).

The relative workload of a given task is lower for individuals with a higher physical capacity (Stevens et al. 2021; Dias et al. 2023). As a natural process of aging, physical capacity, specifically aerobic capacity, declines with age (Boss and Seegmiller 1981; Hamberg-Van Reenen et al. 2009). Consequently, if the physical demands at work are maintained, more employees in physically demanding professions will be expected to work with a higher relative cardiovascular load as they age (de Zwart et al. 1995; Merkus et al. 2019; Stevens et al. 2021). Occupations in the construction industry, commercial kitchens, cleaning, and healthcare are generally considered physically demanding occupations with a high prevalence of musculoskeletal disorders (Haukka et al. 2012; Woods and Buckle 2006; Umer et al. 2018). There are however only a few studies using objective measures of occupational cardiovascular workload in these occupational groups (Korshøj et al. 2015; Lidegaard et al. 2018; Merkus et al. 2019; Dias et al. 2023).

To increase knowledge on the physical demands of senior workers in physically demanding occupations, This study aimed to 1) investigate the cardiovascular fitness among older (50 +) employees; 2) measure and determine the mean occupational cardiovascular load at work; and 3) explore the variation in cardiovascular work intensities in four physically demanding work sectors, namely the construction industry, commercial kitchen work, cleaning of premises, and assistant nurses in health- and elderly care. The study was performed by using data from the High Physical Exposure Study (HIPE-study).

## Methods

### Recruitment and study participants

Workers from the four work sectors were recruited, included, and measured in the study between August of 2021 and April 2023. In the first step, workplace representatives such as human resources personnel or managers were contacted to anchor the organizational interest in joining the study and to approve employees to participate during work hours. Thereafter, employees were informed about the study. Communication strategies differed between organizations, with combinations of written information, information via managers, and verbal information where researchers informed about the study at workplace meetings. To be included in the study, participants should be 50 years or older, work more than 20 h per week, and work full-day work hours when working. A total of 129 workers were primarily recruited for this study, but three were excluded since they had heart disease that was assessed to interfere with the validity of the technical HR measurements (Sammito et al. 2024). Of these three, one person had atrial fibrillation, one had arrhythmia with bradycardia and heart failure, and one had established ischemic heart disease with low work capacity at the exercise test. This left us with 126 recruited participants. Of these, 35 were construction workers from 12 organizations in the private sector. The construction workers included 18 carpenters, seven painters, seven floor layers, two concrete workers, and one smith. Kitchen workers (n = 36), of which 26 cooks and 10 kitchen assistants, were recruited from eight organizations, both publicly funded and from the private sector. Cleaners (n = 29) were recruited from five publicly funded organizations, such as municipalities, regional healthcare, or universities. Assistant nurses (n = 26) were recruited from labor-intensive care units in the regional healthcare organization and from two municipalities, all of them publicly funded organizations.

The HIPE-study received ethical approval from the Swedish Ethical Review Authority (Dnr 2020-01927, 2020-05850 and 2021-03400). All participants signed an informed consent form for the health investigation, questionnaires and technical measurements performed in the study.

### Data collection

All included participants underwent a health examination performed by experienced research nurses. At the health examination, participants reported if they had diseases diagnosed by a doctor like hypertension, asthma, atrial fibrillation, or diabetes, and in those cases, what medicines they were using. Participants also filled out a questionnaire about their work environment and health. Following the health examination, measurements of the cardiovascular load were performed by measuring HR during three working days.

#### Health examination

The participants were asked to refrain from nicotine, coffee, and tea prior to the health examination and to avoid heavy exercise and stress the day before the examination. The participants’ height was measured without shoes using a digital stadiometer (Seca 264, Germany) with an accuracy of 0.1 cm. Body weight was measured with a digital scale (Seca 861, Germany) with 0.1 kg accuracy, wearing light clothing or underwear. Body mass index (BMI) was calculated using the equation BMI = weight (kg)/height (m)^2^. Waist circumference was measured with a measuring tape placed midway between the top of the iliac crest and the lower edge of the last palpable rib, with 0.5 cm accuracy. Blood pressure was measured on the right arm using a blood pressure monitor (Omron M6, Japan) with 0.5 mmHg accuracy when the participant had been sitting down for five minutes. The mean value from two blood pressure measurements was registered. Resting HR was measured with an HR monitor (POLAR, Finland) as the lowest recorded HR during ten minutes of lying rest.

Aerobic physical capacity tests followed the standard test procedure for Ekblom-Bak’s submaximal cycle ergometer test (Ergomedic 828 E, Monark AB, Varberg, Sweden) (Ekblom-Bak et al. 2014). The test has proven to be valid for adults (Väisänen et al. 2020, 2024).

#### Questionnaire

From the questionnaire, we used measures of age, gender, occupation, blood pressure medication, tobacco use, self-rated health, leisure-time exercise, and perceived work ability. Smoking status was assessed with the question “Do you smoke?” and the response alternatives “yes”, “no, have never smoked,” and “former smoker.” Self-rated general health was assessed with the question “In general, you would say that your health is…” with five response alternatives between “excellent”, and “poor” (Orwelius et al. 2018; Hays and Morales 2001). Leisure-time exercise was assessed with the question “How often have you worked out or exercised in training clothes during the last 3 months, with the purpose of improving your physical capacity and/or your well-being?”, with five response alternatives from “never”, to “more than three times per week” (Ng et al. 2011). Work ability was assessed using the Work Ability Score: “How is your current work capacity compared with when it was at its best during your lifetime?”, on a 11-graded scale from “completely unable to work” to “work ability at its best right now” (Ilmarinen 2006; Ahlstrom et al. 2010).

### Assessment, data processing, and statistical analysis of cardiovascular load at work

Measurements of cardiovascular load at work were conducted using a chest band equipped with an electrocardiogram (ECG) driven pulse sensor (Movesense Ltd, Vantaa, Finland; running firmware version 2.0). The sensor recorded HR and RR intervals, and transmitted the data to a mobile application, Wergonic V1.0.7, via Bluetooth (Wergonic AB, Stockholm, Sweden). Study participants wore the HR sensor on three consecutive full working days. Movesense sensors report both HR and RR intervals; the RR interval was used since the reported HR is an average of several beats. To mitigate sensor-related limitations, data preprocessing was performed by removing values reported as zero or excessively high, at the start and end of recordings; subsequently, ectopic beat detection was conducted using a 20% filter, and these intervals were subsequently excluded. The analysis of cardiovascular load during work included several metrics to assess physical strain and recovery periods. The data output for cardiovascular load includes: 1) Mean HR of the working day, 2) Mean %HRR of the working day, 3) Mean HR from the most strenuous hour during the working day, 4) Mean HR from the lightest hour during the working day, and 5) HR peak load (95 percentile). Relative heart rate reserve (%HRR) was calculated using the following equation:

$${\text{\% HRR at work}} = { }\frac{{\left( {{\text{HR}}_{{{\text{work}}}} - {\text{HR}}_{{{\text{min}}}} } \right)}}{{\left( {{\text{HR}}_{{{\text{max}}}} - {\text{HR}}_{{{\text{min}}}} } \right)}} \times 100 $$

HR_work_ was the mean HR during the working day. Maximum HR (HR_max_) was given by the widely used equation 208 − 0.7 × age (Tanaka et al. 2001; Dias et al. 2023). For minimum HR (HR_min_), the lowest measurement of the following was used: 1) the resting HR measured at the health examination; or 2) the lowest HR from the working day measurement, with a running 15-s window. The mean values from the “most strenuous hour” and “lightest hour” offer insights into the variation of the cardiovascular load and recovery periods within the working day and were determined by identifying the maximum and minimum of the smoothed signal obtained using a 1-h moving average. Further, we present the relative time spent in different intensity zones and the minutes per day spent in the different intensity zones. The quantification of absolute and relative time spent across different heart rate intensities included time spent: 1) below 20% of HRR; 2) 20–29% of HRR; 3) 30–39% of HRR; 4) 40–49% of HRR; and 5) 50–69% of HRR. To prevent measurement bias and still use as much of the collected data as possible, we calculated a mean value for each individual across his/her days of measurements, which was included in the overall group mean. When analyzing the number of workdays over different thresholds, we used all available measurement days. The absolute time spent in different heart rate intensities was also calculated to correspond to an eight-hour working day.

The data processing was conducted using MATLAB 2023a (MathWorks, Inc., USA), incorporating scripts from the open-source project HRVAS (Heart Rate Variability Analysis Software) (Ramshur 2010). In the analysis, the first six hours of measurement data for each working day were used. To be included in the analysis, the working day should have at least 300 min of valid data after data processing. Time spent in breaks was included in the measurements. The variables for the HR data were normally distributed. Descriptive statistical analysis was performed using IBM SPSS 27.0 (IBM Corporation, New York, USA). We used the Kruskal Wallis test to analyze differences between work sectors regarding cardiovascular fitness. For this test we used the 7-graded scale based on gender- and age-specific reference values for the Ekblom-Bak test (Väisänen et al. 2024).

## Results

Of the 126 participants, six were excluded from the analysis due to missing HR data. Three of them did not perform HR measurements, of whom two were due to sick leave at the time point for the planned measurements, and one found the chest band uncomfortable to wear. Three of the participants had missing HR data for technical reasons. The final study sample consisted of 120 participants with valid HR data eligible for analysis.

The final study population included 63% women, with a variation between work sectors. The mean age was 50–67 years (mean 57 years), and had a mean BMI of 26.9 (SD 4.4). Of the participants, 10 had diabetes, 12 had asthma, and 40 had a diagnosis of hypertension. Forty of the participants were using blood pressure medication, and 10 used beta-blockers. Participants are further described in Table 1. Three of the participants did not complete the submaximal cycle test due to medical conditions that were identified during the examination. The average VO_2_max was 33.2 (SD 8.2) ml/kg/min for all 117 participants who completed the submaximal tests, for men 39.0 (SD 7.0), and for women 29.8 (SD 6.9). When applying the gender- and age-specific reference values for the Ekblom-Bak test, 49% of the participants had a low fitness level (very low, low, or somewhat low). The results varied by work sector, and 31% in the construction industry, 47% of the kitchen workers, 69% of the cleaners, and 52% of the assistant nurses had a low fitness level. More detailed information on the distribution of the cardiovascular fitness tests can be found in Supplementary Fig. 1. A significant difference in cardiovascular fitness (ml/kg/min) was observed between the construction workers and the cleaners (*p* = 0.009).

**Table 1:** Background characteristics of the study population (n = 120)

	All n = 120	Construction workers n = 32	Kitchen workers n = 35	Cleaners n = 28	Assistant nurses n = 25
Age (years), mean (SD)	57 (4.1)	56 (3.6)	57.9 (4.3)	57.8 (4.2)	56 (3.9)
Female, n (%)	76 (63)	0 (0)	27 (77)	25 (89)	24 (96)
BMI (kg/m2), mean (SD)	26.9 (4.4)	26.1 (2.5)	26.7 (4.0)	28.0 (5.4)	26.9 (5.6)
Waist circumference (cm), mean (SD)	93 (13)	95 (8)	91 (12)	95 (16)	91 (15)
Systolic blood pressure (mmHg), mean (SD)	138 (18)	142 (18)	138 (18)	135 (20)	135 (16)
Diastolic blood pressure (mmHg), mean (SD)	85 (10)	86 (10)	83 (10)	84 (11)	87 (8)
Blood pressure medication, n (%)	40 (33.3)	14 (38.9)	10 (27.0)	10 (35.7)	7 (26.9)
Beta-blockers, n (%)	10 (8.7)	4 (13.9)	2 (5.4)	2 (7.1)	2 (7.7)
Resting heart rate at health examination, mean (SD)	69 (11)	65 (10)	69 (10)	71 (12)	70 (11)
Cardiorespiratory fitness* (l/min), mean (SD)	2.5 (0.7)	3.2 (0.4)	2.3 (0.6)	2.2 (0.6)	2.1 (0.3)
Cardiorespiratory fitness* (ml/kg/min), mean (SD)	33.2 (8.2)	39.4 (6.7)	31.6 (6.8)	30.2 (8.9)	30.7 (7.3)
Currents smokers, n (%)	6 (5)	2 (5)	2 (6)	0 (0)	2 (8)
Current snus users, n (%)	21 (18)	11 (30)	5 (14)	4 (14)	3 (12)
Work ability (grade 0–10), mean (SD)	7.5 (1.6)	7.2 (1.3)	7.7 (1.4)	7.7 (1.5)	7.3 (2.0)
*General health, n (%)*					
Poor or fair	33 (28)	9 (28)	9 (26)	9 (32)	6 (25)
Good	65 (55)	20 (63)	20 (57)	14 (50)	11 (46)
Very good or excellent	21 (18)	3 (9)	6 (17)	5 (18)	7 (29)
*Leisure time exercise, n (%)*					
Never	43 (36)	14 (46)	12 (34)	11 (39)	6 (25)
Once a week or less	38 (32)	7 (22)	10 (29)	10 (36)	12 (46)
Twice a week or more	38 (32)	11 (32)	13 (37)	7 (25)	7 (29)

*Results based on 117 participants performing the aerobic capacity test

**Fig. 1:**
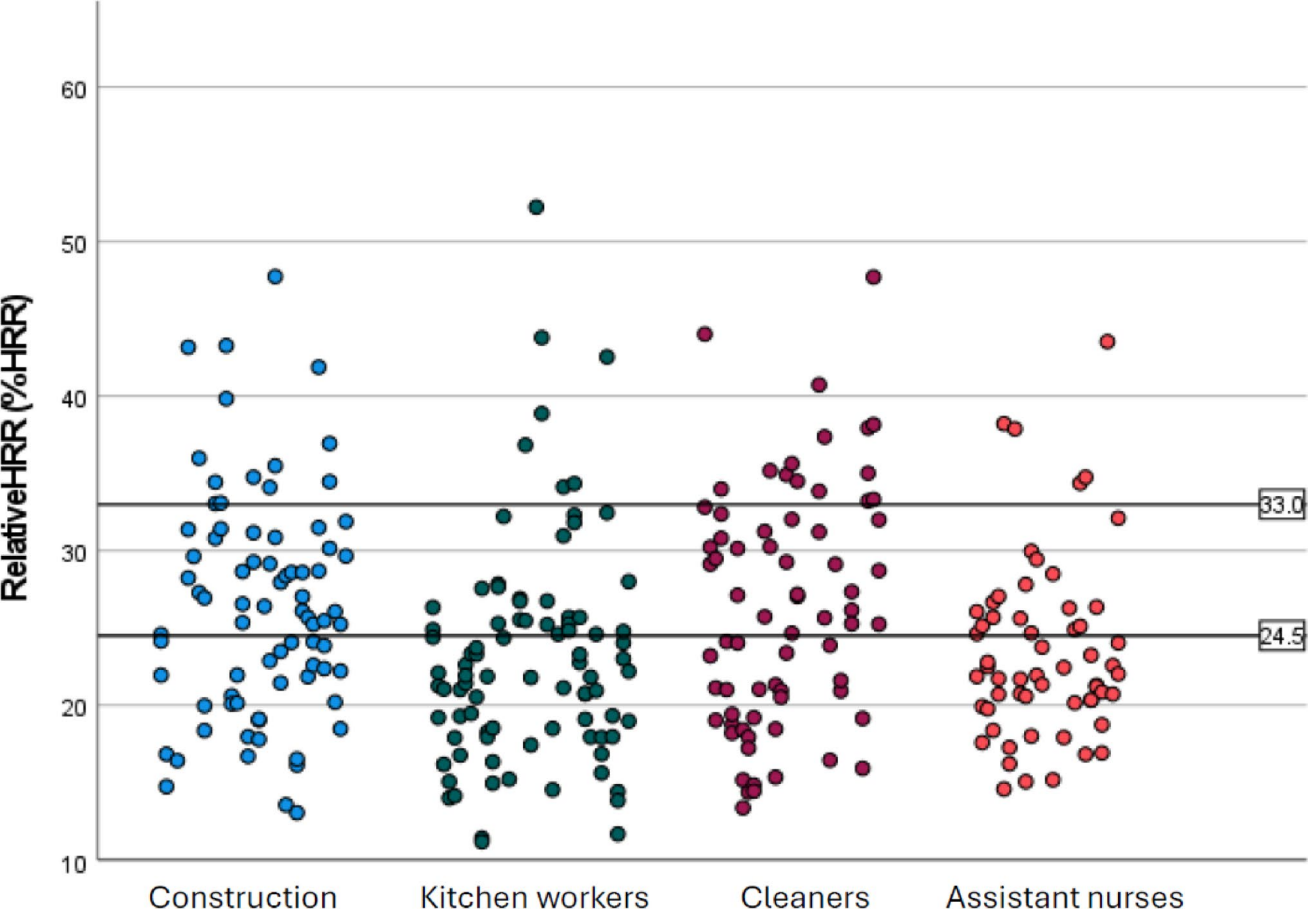
Relative HRR (%HRR) for all 296 measured workdays, illustrated by work sector. The black vertical lines represent 24.5%HRR and 33%HRR

There were a total number of 296 valid measurement days, which corresponds to 2.5 days of valid HR data per participant, with an average length of 359 (SD 4.2) minutes. Of the participants, 27 had eligible data from 1 measurement day, 12 had data from two days, 79 had data from three days, and two had data from four days. The mean %HRR for all occupational groups was 24.9 (SD 6.8) (Table 2). When mirroring our results against previously suggested and reported thresholds to define heavy physical load, 24.5 and 33%HRR, respectively, 43% of the workers had an average load above 24.5%HRR and 11% above 33%HRR (Figure 1 and Supplementary Table 1). When analyzing all the measured days separately, a total of 47% of measured days were above 24.5%HRR and 14% above 33%HRR.

**Table 2:** Cardiovascular load at work, based on 6-h measurements with more than 5 h of valid heart rate data

	All, n = 120	Construction workers n = 32	Kitchen workers n = 35	Cleaners n = 28	Assistant nurses n = 25
Number of measured days, n	296	78	88	71	59
Measured day/worker	2.5	2.4	2.5	2.6	2.4
Mean HR at work, mean (SD)	89 (11)	90 (12)	87 (12)	91 (11)	89 (10)
Mean %HRR, mean (SD)	24.9 (6.8)	26.5 (7.1)	22.8 (6.7)	26.2 (6.9)	24.5 (5.6)
HR for the most strenuous hour, mean (SD)	97 (14)	97 (14)	95 (15)	99 (14)	97 (11)
HR for the lightest hour, mean (SD)	82 (11)	81 (12)	80 (12)	82 (10)	83 (11)
HRpeak, 95 percentile, mean (SD)	105 (12)	107 (13)	102 (14)	106 (12)	104 (9)
Minutes per 8-h workday spent in different intensities of %HRR, based on 6-h measurements with more than 5 h of valid heart rate data					
0–19%, mean (SD)	179 (100)	161 (99)	216 (103)	155 (107)	177 (76)
20–29%, mean (SD)	153 (49)	142 (38)	152 (51)	156 (50)	165 (56)
30–39%, mean (SD)	96 (55)	112 (53)	74 (54)	102 (66)	96 (34)
40–49%, mean (SD)	38 (45)	46 (45)	25 (34)	51 (49)	33 (50)
50–69%, mean (SD)	14 (32)	20 (38)	12 (35)	15 (26)	9 (20)

The mean value for each individual across measurement days was used to calculate group means

**Fig. 2:**
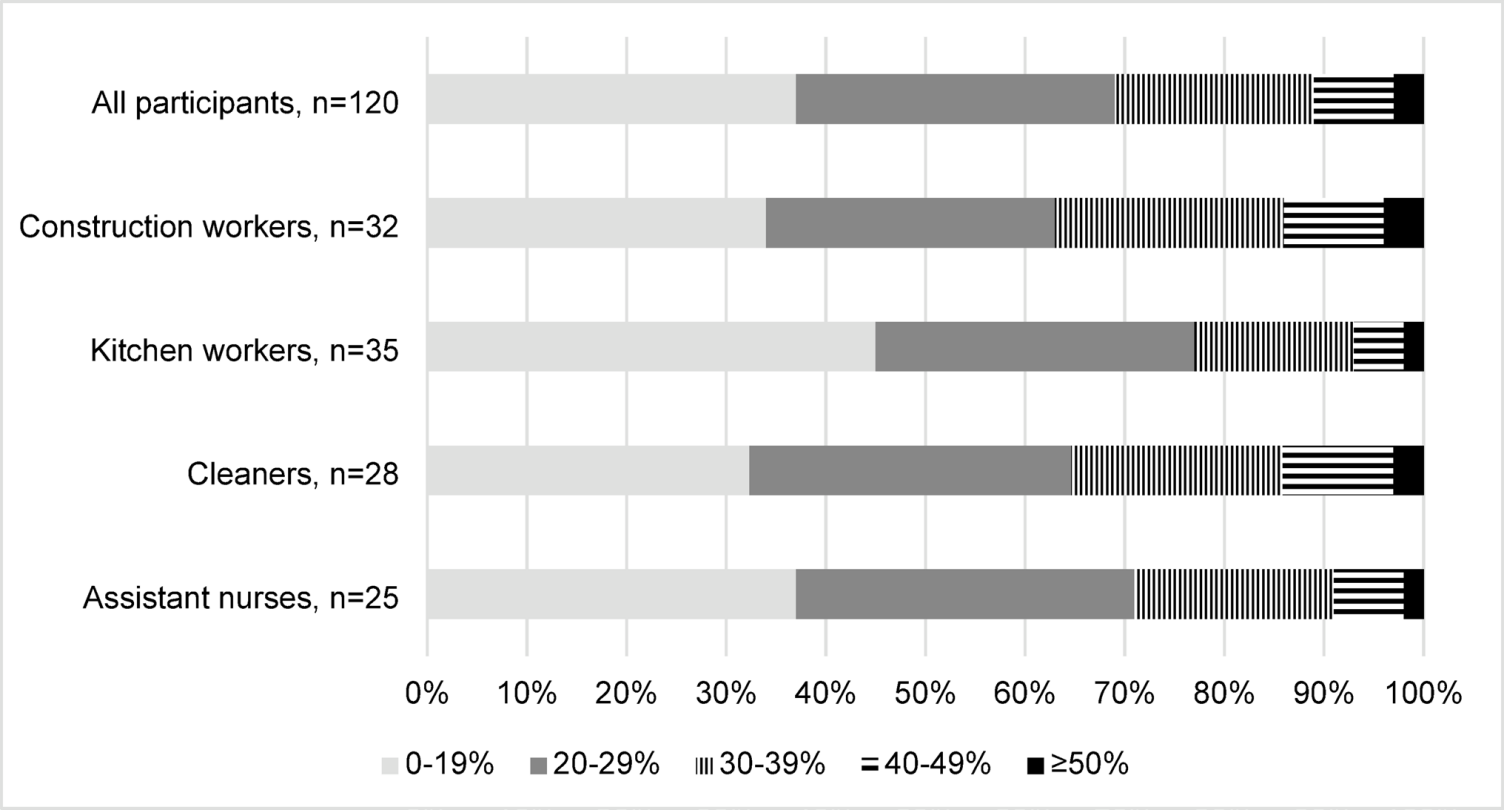
Relative time of the workday spent in different heart rate intensities for all participants and for the separate work sectors

Figure 2 shows the relative distribution of time spent in different heart rate intensities during a working day for the entire population and for those in different work sectors. Workers had a cardiovascular load over 30%HRR between 23 and 37%, and a cardiovascular load over 40%HRR between 7 and 14% of the working days depending on the work sector. For all groups, the average time over 40%HRR was 52 min per day, varying between work sectors, with construction workers and cleaners spending 66 min, while kitchen workers and assistant nurses spending 37 and 42 min per working day over 40%HRR, respectively (Table 2). Depending on the work sector, between 0.2 and 0.8% of the measured time exceeded the intensity of 60%HRR. This equals 1–4 min per eight-hour working day (Supplementary Table 1).

## Discussion

In the current study, we aimed to quantify the occupational cardiovascular load among older employees in occupations with high physical demands. Our results display a relatively high cardiovascular load at work, averaging 25%HRR.

We found cleaners to have an average cardiovascular load of 26%HRR, which is in the same range as previous studies in cleaners who reported 24–30%HRR (Korshøj et al. 2013, 2015; Balogh et al. 2004). In the construction industry and healthcare sector, Merkus et al. (2019) found older construction and healthcare workers over 45 years to have %HRR values of 29 and 25.5, respectively, which was slightly higher than our results. Compared with their study population, our participants were older and included more homogeneous occupational groups. Further, studies in cleaners and healthcare workers (Balogh et al. 2004; Merkus et al. 2019) found older workers to have a higher relative %HRR compared with younger colleagues, while the opposite was found in construction workers (Merkus et al. 2019). This might suggest that older workers in female-dominated sectors have a higher aerobic workload compared with their younger colleagues, which might be due to working with the same work tasks and absolute demands, leading to a higher relative cardiovascular load. Older workers in the construction sector might have a higher possibility of shifting to tasks with lower absolute physical demands or could perform the tasks with a different style, compared to their younger colleagues. Compared with previous studies among cleaners with recruitment in the corresponding organizations, our participants were older. To the best of our knowledge, there are no previous studies investigating the cardiovascular load in kitchen workers using technical measurements to compare our findings. The variations between individuals likely reflect differences in factors such as work tasks, work style, and fitness level, leading to varying levels of physical effort.

A previous study that performed measurements of oxygen uptake on older construction workers reported that workers spent most of their time below 30% of their maximum oxygen uptake, but short periods of higher intensity were observed on some tasks (Jebens et al. 2015). Wu and Wang (2002) suggest that an oxygen uptake of 33% corresponds to 24.5%HRR. Given that 23–37% of the work time in the current study was spent over 30%HRR, our findings are consistent with those of Jebens. However, the relation between %VO_2_ and %HRR is not a universal relationship since it is influenced by for example age and fitness, which could vary between study populations. The observed relationship when comparing the different values could also be influenced by the measurement equipment used (Van Hooren et al. 2024). Other studies have used 40%HRR as a limit to describe high occupational physical strain, and a cohort study in blue-collar workers found male versus female workers to spend 17 and 11% of their working day above 40% HRR, respectively (Rasmussen et al. 2019). In line with this, we observed that the workers in the construction industry and the cleaners spent 14% of the working day over 40%HRR, whereas kitchen workers and assistant nurses differed, spending 7% and 9% of the working day above 40%HRR, respectively. In summary, we found the four studied occupational groups to be highly physically demanding. The high demands might lead to insufficient recovery and permanent overstraining with negative health consequences and work-related disability (Hallman et al. 2017; Holtermann et al. 2018; Gupta et al. 2020; Korshøj et al. 2021; Merkus et al. 2022). Further, the high physical workloads are likely barriers to prolonging working life in these industries (Kadefors et al. 2018).

Compared with a reference working population, almost half of the participants in our study had a low aerobic physical capacity (Väisänen et al. 2024). These findings follow previous research on older workers in the construction industry and in the healthcare sector (Jebens et al. 2015; Merkus et al. 2019) while younger cleaners in a Danish study had a higher capacity compared with our group (Lidegaard et al. 2018). To achieve an increase in aerobic capacity, the intensity and duration of the PA are important, and the time for recovery needs to be sufficient. Despite the relatively high average workload, we found that only a few minutes per working day was spent in heart rate intensities that could be expected to effectively increase cardiorespiratory fitness (above 60%HRR) (Garber et al. 2011). We found that construction workers spent up to 4 min per day above 60%HRR, whereas the other groups spent even less time in higher intensities. Lunde et al (2016) also found construction workers to spend low amounts of time in higher intensities during work and showed that the occurrence of continuous episodes of higher intensity was rare. On the other hand, studies on high-intensity interval exercise with short intervals have shown to be effective in increasing aerobic capacity (Simonsson et al. 2023). The occurrence of higher peak loads in the construction workers might explain why there was a lower proportion of construction workers with low cardiorespiratory fitness despite reporting less leisure time exercise. Our findings follow results in previous studies and confirm the proposed physical activity paradox, suggesting that OPA in these occupations is inadequate to increase physical fitness and might instead have detrimental effects on health (Holtermann et al. 2018). Our results also indicate that more studies evaluating the time spent in various intensities can be advantageous for both understanding cardiovascular risks and designing targeted interventions.

Adjusting and lowering the physical work demands or increasing workers’ individual fitness levels are two theoretical ways to decrease the relative load of workers. Among cleaners, it has been shown to be difficult to decrease the load by ergonomic interventions (Haukka et al. 2008), while an intervention to improve aerobic capacity has been shown to decrease the relative cardiovascular load, reduce the need for recovery, and increase productivity, especially in younger workers (Korshøj et al. 2015; Lidegaard et al. 2018). Further, Holtermann et al. (2009) found that high OPA had detrimental health effects with increased ischemic heart disease mortality in unfit workers, but not among fit personnel. Even though exercise interventions could be a way forward, the increasing prevalence of chronic diseases might be a complicating factor regarding exercise interventions in older workers. An intervention study aiming to increase fitness in cleaners found that participants with more than 30%HRR at work showed negative intervention effects on ambulatory and resting blood pressure (Korshøj et al. 2016). Since cardiorespiratory fitness in Swedish adults seems to be declining, especially in groups with low educational levels (Väisinen et al. 2021), there is a growing need to improve and maintain aerobic capacity in workers of all ages, both from public health and employer perspectives. Based on an increased vulnerability to aging and the fact that younger individuals seem to gain stronger effects in exercise interventions, these health-promoting measures should be introduced early in working life (Lidegaard et al. 2018). In addition to managing the physical demands of these occupations, psychosocial factors are also crucial in facilitating and encouraging extended working lives (Nilsson 2012, 2016). Collegial support, encouragement, and opportunities for development have been shown to positively influence retirement age (Andersen et al. 2024).

Depending on methodological decisions for how calculations of relative cardiovascular load are performed, results from studies may differ. Different threshold variables have been used to define limits for harmful occupational cardiovascular strain, which complicates study comparisons and makes it difficult to establish guidelines for working life (Dias et al. 2023). In this study, we have reported and discussed limit values of 30 and 33%HRR in relation to previous research. However, since %HRR does not directly correspond to %VO_2_ max, we also used a limit value of 24.5%HRR, which, according to Wu and Wang (2002), corresponds to 34%VO_2_ max during an eight-hour working day. In our study, 47% of the measured working days had an average cardiovascular load above 24.5%HRR and 14% above 33%HRR. These results exemplify how the definition of thresholds impacts the interpretation of results, and we suggest the use of 24.5%HRR in future studies. However, more studies on different populations and particularly on elderly workers are needed to strengthen the knowledge of relevant and reliable exposure thresholds in terms of %VO_2_ max and corresponding %HRR during a working day.

Methods for measuring resting HR also differ, which affects the calculation of cardiovascular load and makes comparisons of results from different studies difficult (Dias et al. 2023). We defined resting HR as the lowest assessment from either the health examination or the lowest HR from the working day measurement, with a running 15-s window. The reason for this approach was that some of the participants had a lower HR measured at work than the resting HR obtained at the health examination. This could be an effect of mental stress in conjunction with the clinical examination compared with a more relaxed situation during breaks at work (Lequeux et al. 2018). In studies using %HRR to establish cardiovascular load, some have defined resting HR from measurements during sleep (Jørgensen et al. 2019; Lidegaard et al. 2018; Rasmussen et al. 2019), while others have used daytime measurements of resting HR (Ismaila et al. 2012; Merkus et al. 2019). HR during sleep is considerably lower compared with resting HR during awake time (Speed et al. 2023), and calculations of %HRR based on resting HR during sleep will lead to higher values for %HRR as compared with daytime measurements of resting HR (Korshøj et al. 2013, 2015).

### Strengths and limitations

The strengths of our study include a relatively large number of participants who consented to the technical data collection over several days. The studied population was homogeneous regarding age and represented different physically demanding occupations since they were recruited from four different work sectors, including many organizations. Further, the metrics used to present the results give a comprehensive overview of the intensity and distribution of cardiovascular load, offering valuable insights into the balance between physical exertion and recuperation over the working day.

Limitations of the study include the risk of recruitment bias, which may give rise to a healthy worker effect, leading to an overestimation of physical capacity and an underestimation of the relative workload. In our study, as well as in previous studies, there are limitations in the use of different exposure thresholds, based on assumptions with built-in flaws regarding the relations between %VO_2_ and %HRR. Another limitation was the use of a submaximal test when estimating the VO_2_max. Even though the submaximal test used in this study is a validated method for the estimation of VO_2_max (Ekblom-Bak et al. 2014; Väisinen et al. 2020), a more exact measure of the VO_2_max would have been determined with a maximal test. However, it is likely that some of the participants would not have signed up for the study if a maximal test had been included.

## Conclusion

In conclusion, we found a high average cardiovascular load among the senior workers in four work sectors with high physical demands. Despite a high cardiovascular load at work, a high proportion of the workers had low cardiovascular fitness. Given the possible negative effect of OPA on health and the need to meet future demographic challenges, future interventions should aim to reduce physical loads and increase physical fitness in the workforce.

## Supplementary Information

Below is the link to the electronic supplementary material.

Supplementary file1 (DOCX 50 kb)

## Data Availability

The data that support the findings of this study are not openly available due to reasons of sensitivity and are available from the corresponding author only upon reasonable request. Data are located in controlled access data storage at Umeå University.
