# Supplementary material for: Cardiovascular load and physical capacity in older workers engaged in physically demanding occupations
Source: Int Arch Occup Environ Health. 2025 Aug 14;98(7):673–83. doi: 10.1007/s00420-025-02161-8 (PMC12484335; doi:10.1007/s00420-025-02161-8)
Supplement: Supplementary file 1 — Supplementary file1 (DOCX 50 kb) [file 420_2025_2161_MOESM1_ESM.docx]

## Supplementary table

| Supplementary table 1. Cardiovascular load at work | | | | | |
| --- | --- | --- | --- | --- | --- |
|  | All  n=120 | Construction workers  n=32 | Kitchen workers  n=35 | Cleaners  n=28 | Assistant nurses  n=25 |
| Relative time spent in different intensity zones, shown in figure 1, based on mean value across measurement days for each individual. | | | | | |
| 0- 20%, mean (SD) | 37 (21) | 34 (21) | 45 (22) | 32 (22) | 37 (16) |
| 20-29%, mean (SD) | 32 (10) | 29 (8) | 32 (11) | 32 (10) | 34 (12) |
| 30-39%, mean (SD) | 20 (11) | 23 (11) | 16 (11) | 21 (14) | 20 (7) |
| 40-49%, mean (SD) | 8 (9) | 10 (9) | 5 (7) | 11 (10) | 7 (10) |
| 50-59 %, mean (SD) | 2 (5) | 3 (6) | 2 (5) | 3 (5) | 2 (4) |
| 60-69 %, mean (SD) | 0.5 (2) | 0.8 (2) | 0.6 (2) | 0.3 (0.7) | 0.2 (0.6) |
| 50-69 %, mean (SD) | 3 (7) | 4 (8) | 2 (7) | 3 (6) | 2 (4) |
| Number of workers with an average load over different %HRR thresholds, based on mean value across measurement days for each individual. | | | | | |
| >24.5 %HRR, n (%) | 52 (43) | 16 (50) | 12 (34) | 16 (57) | 8 (32) |
| >30 %HRR, n (%) | 24 (20) | 10 (31) | 4 (11) | 8 (29) | 2 (8) |
| >33 %HRR, n (%) | 13 (11) | 5 (16) | 2 (6) | 4 (14) | 2 (8) |
| Number of workdays with an average load over different %HRR thresholds, based on all 296 valid measurement days. | | | | | |
| >24.5% HRR, n (%) | 140 (47) | 46 (59) | 32 (36) | 40 (56) | 22 (37) |
| >30% HRR, n (%) | 65 (22) | 22 (28) | 12 (14) | 25 (35) | 6 (10) |
| >33% HRR, n (%) | 41 (14) | 14 (18) | 7 (8) | 15 (21) | 5 (9) |


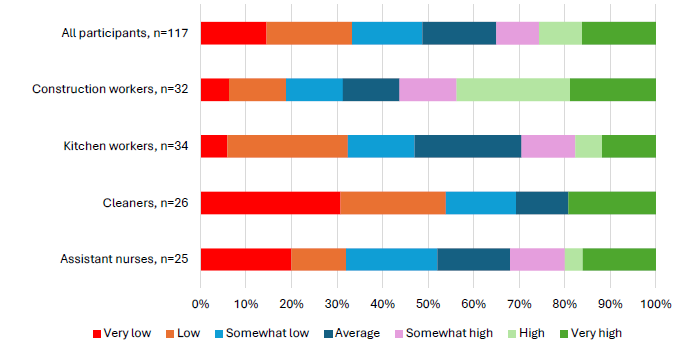


Supplementary figure 1. Distribution of the cardiovascular fitness test results for all participants and for the individual work sectors, presented in relation to reference values (Väisinen et al. 2024).
